# Supplementary material for: Characterizing measles transmission in India: a dynamic modeling study using verbal autopsy data
Source: BMC Med. 2017 Aug 10;15:151. doi: 10.1186/s12916-017-0908-3 (PMC5550950; doi:10.1186/s12916-017-0908-3)
Supplement: Additional file 1: — Web appendix I: Million Death Study research ethics. Web appendix II: Model structure. Web appendix III: Parameter estimation. (PDF 17 kb) [file 12916_2017_908_MOESM1_ESM.pdf]

Verguet *et al. BMC Medicine* _####################_
DOI 10.1186/s12916-017-0908-3

BMC Medicine

RESEARCH ARTICLE Open Access

CrossMark

Characterizing measles transmission in India: a dynamic modeling study using verbal autopsy data

Stéphane Verguet[1*†], Edward O. Jones[2†], Mira Johri[3,4], Shaun K. Morris[5], Wilson Suraweera[6], Cindy L. Gauvreau[7], Prabhat Jha[6] and Mark Jit[2,8]

Abstract

Background: Decreasing trends in measles mortality have been reported in recent years. However, such estimates of measles mortality have depended heavily on assumed regional measles case fatality risks (CFRs) and made little use of mortality data from low- and middle-income countries in general and India, the country with the highest measles burden globally, in particular.

Methods: We constructed a dynamic model of measles transmission in India with parameters that were empirically inferred using spectral analysis from a time series of measles mortality extracted from the Million Death Study, an ongoing longitudinal study recording deaths across 2.4 million Indian households and attributing causes of death using verbal autopsy. The model was then used to estimate the measles CFR, the number of measles deaths, and the impact of vaccination in 2000–2015 among under-five children in India and in the states of Bihar and Uttar Pradesh (UP), two states with large populations and the highest numbers of measles deaths in India.

Results: We obtained the following estimated CFRs among under-five children for the year 2005: 0.63% (95% confidence interval (CI): 0.40–1.00%) for India as a whole, 0.62% (0.38–1.00%) for Bihar, and 1.19% (0.80–1.75%) for UP. During 2000–2015, we estimated that 607,000 (95% CI: 383,000–958,000) under-five deaths attributed to measles occurred in India as a whole. If no routine vaccination or supplemental immunization activities had occurred from 2000 to 2015, an additional 1.6 (1.0–2.6) million deaths for under-five children would have occurred across India.

Conclusions: We developed a data- and model-driven estimation of the historical measles dynamics, CFR, and vaccination impact in India, extracting the periodicity of epidemics using spectral and coherence analysis, which allowed us to infer key parameters driving measles transmission dynamics and mortality.

Keywords: Measles, Vaccine-preventable diseases, Child health, Immunization, Case fatality risk, Supplementary immunization activities, Mathematical modeling, India

Background

The World Health Assembly officially endorsed in 2005 a goal to reduce measles-related deaths by 90% globally between 2000 and 2010 [1]. The Measles & Rubella Initiative (www.measlesrubellainitiative.org), a consortium of leading global health agencies launched in 2001, has been supporting the World Health Organization (WHO) strategies required to ~~achieve~~ measles mortality ~~reduction~~ [2], including routine immunization of children, and supplementing it by a ~~recommended~~ second dose opportunity for measles vaccine [3, 4]. In countries with routine immunization systems that have achieved good population coverage, the second dose of measles vaccine is usually included in the vaccination schedule ~~and administered to children before school entry~~ [4]. Conversely, in countries where routine immunization services have not yet met these targets, ~~an opportunity for a~~ second dose of measles vaccine tends to be ~~offered~~ through supplemental immunization activities (SIAs) [4].

* Correspondence: verguet@hsph.harvard.edu
†Equal contributors
[1]Department of Global Health and Population, Harvard T.H. Chan School of Public Health, 665 Huntington Avenue, Boston, MA, USA
Full list of author information is available at the end of the article

BioMed Central

© The Author(s). 2017 **Open Access** This article is distributed under the terms of the Creative Commons Attribution 4.0 International License (http://creativecommons.org/licenses/by/4.0/), which permits unrestricted use, distribution, and reproduction in any medium, provided you give appropriate credit to the original author(s) and the source, provide a link to the Creative Commons license, and indicate if changes were made. The Creative Commons Public Domain Dedication waiver (http://creativecommons.org/publicdomain/zero/1.0/) applies to the data made available in this article, unless otherwise stated.

Researchers at the WHO have reported decreasing trends in measles mortality [5]. Simons and colleagues [6] estimated that global measles-related deaths decreased from 535,000 (95% confidence interval (CI): 347,000–976,000) in 2000 to 139,000 (71,000–448,000) in 2010. Measles mortality was estimated to have reduced by more than 75% in all regions but the WHO Southeast Asia region [6]. In this region, India accounted for about 50% of global measles mortality in 2010 [6]. Yet, the WHO estimates depended heavily on measles case fatality risks (CFRs) reviewed from the literature spanning more than two decades [7]. Such reviewed CFRs were drawn from a variety of outbreak situations, surveillance methods, reporting conditions, and locations (e.g., community vs. in hospitals), leading to great heterogeneity and vast confidence intervals, and were subsequently pooled into regional estimates [7] for use in WHO's mortality estimation [5, 6]. These estimates made little use of measles mortality data from low- and middle-income countries in general, particularly regarding India, the country with the highest measles burden globally. For instance, available measles data for India include not only national case notifications but also mortality data such as those from the Million Death Study [8–10]. The Million Death Study is an ongoing longitudinal study recording deaths across 2.4 million Indian households (14 million individuals) and attributing causes of death using verbal autopsy. Notably, the observed periodicity in measles deaths, similar to the periodicity in measles cases, can help us identify some of the intrinsic features of measles transmission in India. The periodicity in measles transmission may be difficult to extract because of multiple frequencies and noise contained in the measles mortality time series. However, the use of mathematical techniques such as the Fourier transform [11], which we implement in this study, is ideal for extracting such periodic features and therefore represents a novel aspect of the methodological approach we develop here and subsequently apply to India.

In this paper, we constructed a dynamic model of measles transmission in India that was empirically inferred from a time series of measles mortality extracted from the Million Death Study. The model was then used to estimate among under-five children: (1) the measles CFR, (2) the number of measles-related deaths, and (3) the impact that routine and SIA immunization has had since 2000. Model development and estimation was also pursued for Bihar and Uttar Pradesh (UP), two states with large populations, high under-five mortality rates [12], relatively low vaccine coverage [13], and the highest numbers of measles-related deaths in the country [10].

Methods

Measles deaths data

We used measles deaths data from the Million Death Study (MDS), an ongoing longitudinal study recording deaths occurring in 2.4 million households across India and attributing causes of death using verbal autopsy [8–10]. Deaths attributed to measles were available over a period of 3 years (2000–2003), and we analyzed weekly occurrence of measles deaths over this 3-year period in India and in the states of Bihar and UP since the day of the first measles death recorded (Fig. 1). We used anonymous secondary data (Additional file 1: Web appendix I provides MDS research ethics details), and the full protocol of the MDS can be consulted online (http://www.cghr.org/project.htm).

Dynamic model of measles transmission

We adapted the Dynamic Measles Immunization Calculation Engine (DynaMICE), an existing dynamic compartmental discrete-time model of measles transmission and vaccination stratified by age previously used to examine measles transmission in high burden countries [14] (see Additional file 1: Web appendix II). As discussed in [14], the model population occupied states representing being susceptible to measles, being infected with measles, and having recovered from measles with lifelong immunity thereafter. The rate at which infection occurred depended on the existing proportion of the population who were already infected, the effective contact rate between different age groups, and a periodic forcing term (using a sine function) representing seasonal changes in measles transmissibility. Effective contact rates and mixing between individuals in different age groups were based on the British arm of the POLYMOD social contact survey [15], as with our previous work [14]. We also simulated measles case importation into the population at a rate of one per week, to mitigate against unpredictable effects on infection dynamics due to tiny fractions of infected and numerical solver accuracy.

Although individuals in the population were categorized according to discrete age classes from 0 to 80 years, a proportion of each age class had their age incremented by one at every time step. Vaccinated individuals were assumed to have a reduced risk of measles infection. Vaccine efficacy was assumed to be 85% for the first dose when vaccinating before age one, 95% after age one, and 98% for two doses, as suggested by a meta-analysis [16]. Vaccines were assumed to be "all or nothing": individuals receiving the vaccine were either completely protected or not at all. We assumed that vaccination gave lifetime protection if it successfully elicited an immune response, and that vaccinating already infected individuals did not increase the rate of infection clearance. The model was programmed using the software R version 3.2.2 (www.r-project.org) ~~and is described in great detail elsewhere [14]~~.

As in our previous study [14], the population age distribution was obtained from government sources [17] along with crude birth rates and death rates to inform initial age-specific population sizes and mortality rates.

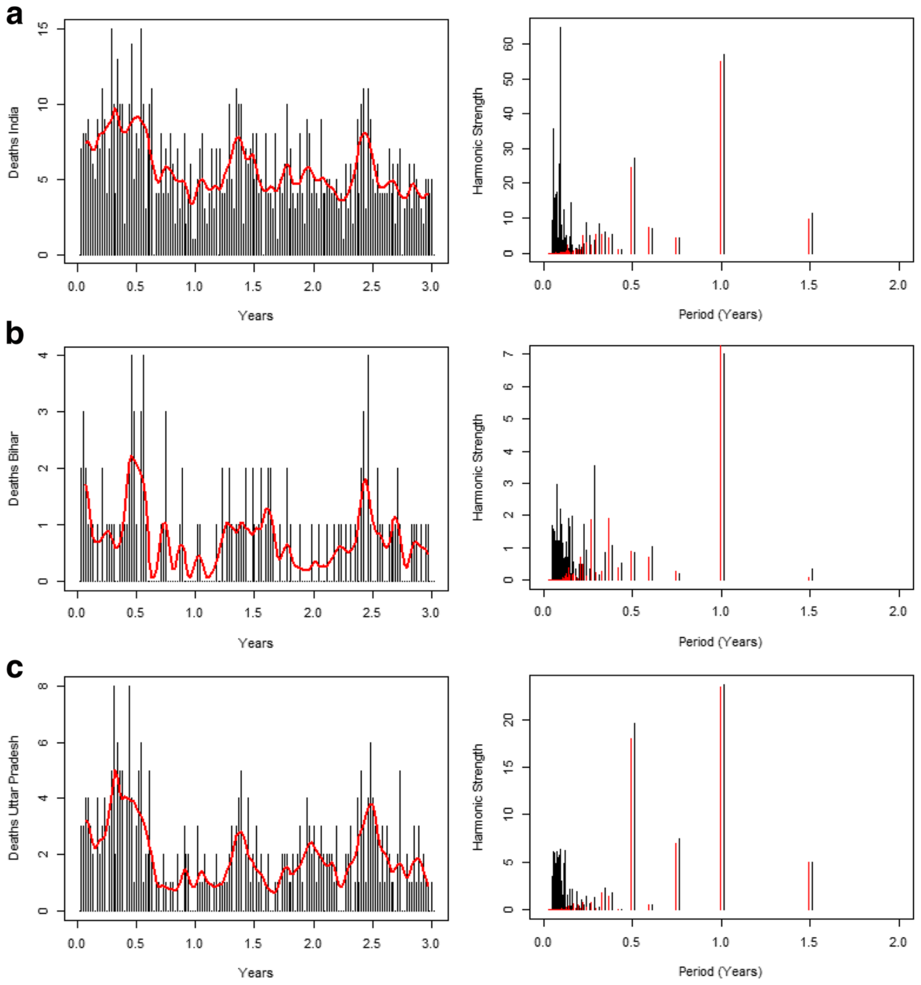

Fig. 1 Weekly measles deaths recorded over the period 2000–2003, for India (**a**), Bihar (**b**), and Uttar Pradesh (**c**), since the day of the first measles death (source Million Death Study), with their associated frequency spectra with strength of harmonic on *y*-axis and period up to 2 years on *x*-axis. The *black lines* correspond to the raw data and corresponding spectra, while the *red line* denotes the data and spectra post-detrending with the Baxter-King filter

These demographic parameters were assumed to be static over time (constant population growth) to avoid difficulties around long-term population projection. The average infectious period of measles was assumed to be 14 days [18]. Routine measles vaccination was assumed to be delivered before age one, with the SIA being given to all children between one and ten years. The probability of receiving a routine and SIA dose was assumed to be uncorrelated. Routine measles vaccine coverage was obtained from government sources [13]. The timing of the rollout and coverage estimates of the SIA doses were based on that achieved by the 2010–2013 measles SIAs [19, 20]. The SIA started in 2010 and increased in coverage year on year over three phases to reach a cumulative coverage estimate of 91% across states receiving the SIA. Different states had different timings; for example, Bihar received the SIA earlier (phases 2 and 3) than UP (phase 3, which started in 2012) [20].

Model calibration

Model dynamics depend primarily on parameters (amplitude of seasonal variation in measles a_0, basic reproduction number R_0) which cannot be directly observed, so we instead infer these parameters by fitting our model to the measles deaths data. First, we used the measles mortality time series (Fig. 1) to infer the periodicity in the oscillations in the number of measles deaths over time in India, Bihar, and UP. To do so, we first detrended the data using a Baxter-King filter [21] and then conducted a spectral analysis of each of the three time series applying a Fourier transform [11]. Subsequently, we obtained a spectrum composed of specific frequencies (or periods) and corresponding amplitudes. In our analysis, we used the five longest periods (1.50, 1.00, 0.75, 0.60, and 0.50 years).

Second, for India, Bihar, and UP, the dynamic model was run for 100 years to allow it to reach a pre-

vaccination equilibrium, then run for a further 100 years with routine vaccination to reach a post-vaccination equilibrium, as implemented in [14]. We assumed routine first dose coverage only (at 56%), as there were no SIAs in the years for which measles mortality data were available (2000–2003). Subsequently, we obtained a periodic time series of measles cases for distinct values of the basic reproduction number (R_0) and the amplitude of the forcing term (a_0). R_0, which was defined using the dominant eigenvalue of the next generation matrix, was varied between 10 and 25 (based on ranges given in [18]), while a_0 was varied between 0 and 0.5 (Additional file 1: Web appendix III, Figure S1). R_0 was set by adjusting the probability of an infected individual transmitting measles to a susceptible individual following an effective contact. The spectrum of the time series of measles cases (once post-vaccination equilibrium was reached) was then extracted for each combination of values of R_0 and a_0. Assuming that periodicity in measles mortality would be the same as periodicity in measles incidence (i.e., a constant CFR), we then compared the model spectrum with the mortality data spectra for India, Bihar, and UP.

We used the coherence function [22] as a measure of similarity between the two time series (Additional file 1: Web appendix III, section 2). Each (R_0; a_0) combination was simulated and run until it reached equilibrium. For each period of interest from the mortality data spectrum, we estimated the coherence between the time series estimated from the (R_0; a_0) combination and the measles mortality time series. By combining the five coherence estimates for each period (1.50, 1.00, 0.75, 0.60, and 0.50 years), we produced a pooled (or combined) coherence [23] for each (R_0; a_0) combination (see the explanation in Additional file 1: Web appendix III), from which we could subsequently calculate a CFR estimate. We also validated our procedure to ensure that a 3-year time series provided sufficient information to recover values of R_0 and a_0 from the underlying infection process (see Additional file 1: Web appendix III, section 2).

Model analysis

First, based on the simulated models obtained, we estimated the total number of measles cases, per given age group, for India, Bihar, and UP for each R_0 and seasonal variation a_0 combination. Subsequently, to estimate CFR, we compared the total numbers of measles cases estimated among under-five children with the numbers of measles deaths among under-five children as extrapolated from the same measles deaths data at the national and state levels for 2005 by Morris and colleagues [10]. If the pooled coherence was significant, we used the associated coherence level for each simulated (R_0; a_0) combination as weight to sample from the estimated cases (i.e., case estimates from an (R_0; a_0) combination that had a coherence value of 0.8 would have twice the probability of being sampled than those with a coherence of 0.4). This method produced a weighted distribution of cases that we could then combine with the sampled distributions of estimated deaths during the same time period [10] (Additional file 1: Web appendix III, Table S1). This yielded a CFR distribution for measles among under-five children for India as a whole, Bihar, and UP, for 2005, provided with 95% CIs embedded in the coherence estimation procedure (Additional file 1: Web appendix III, section 2). Second, using the R_0 and a_0 parameters that produced the greatest pooled coherence across the five chosen periods and the estimated CFRs, we derived the number of measles deaths which would have occurred among under-five children over the period 2000–2015 in India, Bihar, and UP. Finally, we estimated the number of measles deaths for under-five children that would occur over 2000–2015 had (1) the SIAs not happened, and (2) routine vaccination not occurred.

Results

The measles frequency spectrum for India showed strong components for periods of 0.5, 1.0, and 1.5 years. The Bihar frequency spectrum had a principal component of 1.0 year, while the UP spectrum had strong components at periods of 0.5 and 1.0 year (Fig. 1). Subsequently, we ran the coherence estimations. Based on the estimations for the pooled coherence (Fig. 2), we extracted the following combinations for R_0 and a_0: $R_0 = 24$ and $a_0 = 0.18$ for India as a whole, $R_0 = 20$ and $a_0 = 0.10$ for Bihar, and $R_0 = 14$ and $a_0 = 0.16$ for UP.

Furthermore, we plotted the pooled coherence against CFR for India, Bihar, and UP (Fig. 3), to display the associated CFR values to the coherence values. This is done because the varying of R_0 and a_0 sets both the periodicity and the number of cases in the model, which determine the coherence and CFR, respectively. With the pooled coherence estimates, we obtained the following estimates of CFRs among under-five children calibrated to the year 2005: 0.63% (95% CI: 0.40–1.00%) for India as a whole; 0.62% (0.38–1.00%) for Bihar; 1.19% (0.80–1.75%) for UP.

Over 2000–2015, assuming CFRs remained constant over time, we estimated that a total of 607,000 (95% CI: 383,000–958,000) under-five deaths attributed to measles occurred in India as a whole. Likewise, 100,000 (61,000–161,000) under-five deaths caused by measles occurred in Bihar, which represented about 17% of all Indian under-five measles deaths; 313,000 (211,000–461,000) under-five deaths caused by measles occurred in UP, which represented around 52% of all Indian under-five measles deaths.

If the SIAs of 2010–2013 had not occurred in India, 66,000 (95% CI: 41,000–108,000) excess under-five

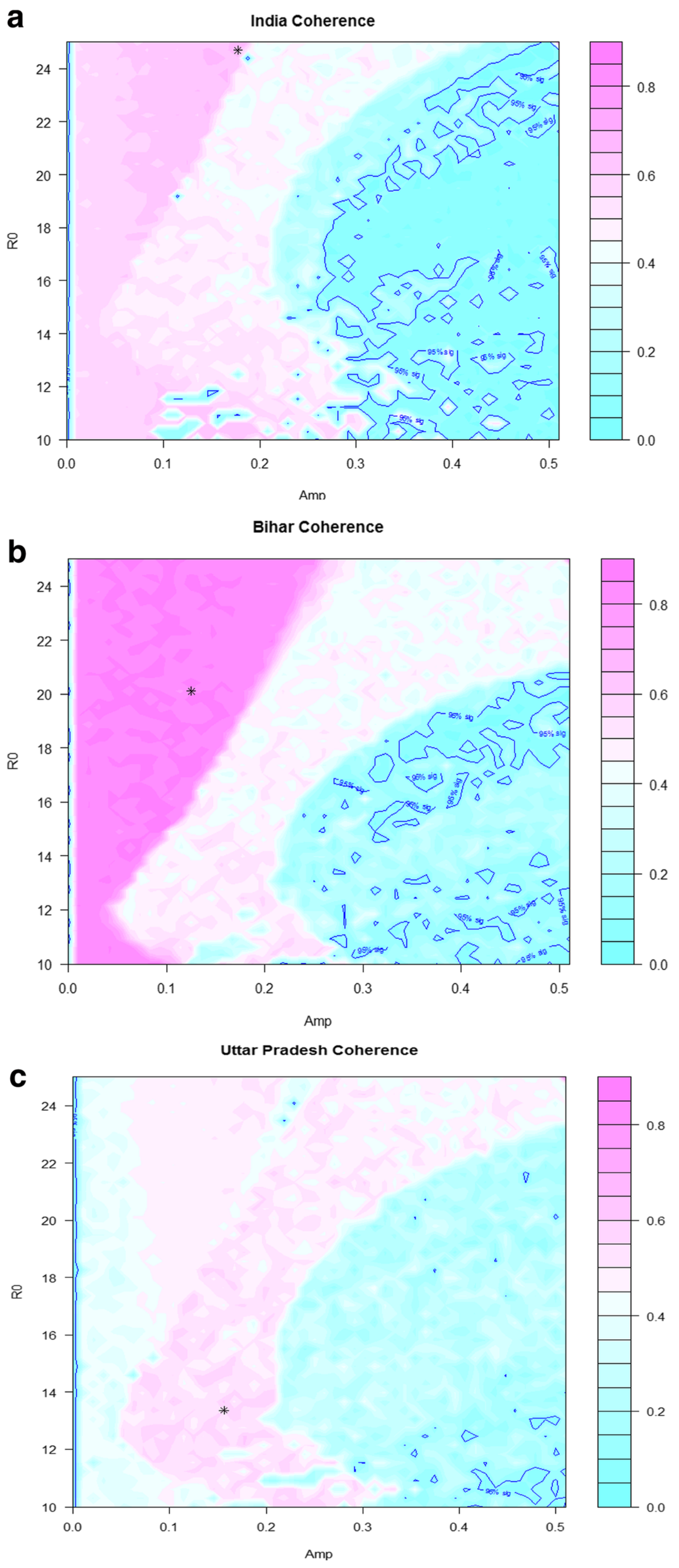

Fig. 2 (See legend on next page.)

(See figure on previous page.)

Fig. 2 Pooled coherence estimate across five periods (0.5, 0.6, 0.75, 1.0, 1.5 years) for India (**a**), Bihar (**b**), and Uttar Pradesh (**c**), plotted against estimated basic reproduction number (R_0) and amplitude of the forcing term (*Amp* or a_0). *Pink* indicates a better match to periodicity in the mortality data. The location of the maximum coherence is identified with an *asterisk* (*), and the 95% significance contour (coherence = 0.11) is marked in *dark blue*

deaths would have occurred; among which 7000 (4100–11,300) would have occurred in Bihar and 40,000 (27,000–58,000) in UP. Lastly, if no routine vaccination coverage or SIA occurred from 2000 to 2015, an additional 1.6 million (95% CI: 1.0–2.6 million) under-five deaths would have occurred across India with 110,000 (70,000–169,000) in Bihar and 491,000 (331,000–724,000) in UP.

Discussion

We present in this paper a data- and model-driven estimation of the historical measles dynamics, CFR, and vaccination impact in India as a whole as well as in two key states (Bihar and UP) for measles mortality. We used measles mortality data from verbal autopsy studies from a nationally representative longitudinal cohort of 2.4 million households and extracted the periodicity of measles epidemics, which allowed us to infer key parameters driving the dynamics of measles transmission. To do this we used spectral and coherence analysis together with statistical inference using an age-stratified dynamic compartmental model of measles transmission. This represents a first-of-a-kind modeling approach for measles transmission in the developing world.

Spectral analysis of measles mortality data suggested that the time series consisted of the superposition of several cycles of measles epidemics with different inter-epidemic periods. We were able to estimate very high basic reproduction numbers ($R_0 > 20$) for the country as a whole and Bihar. The case of UP with its lower estimated basic reproduction number ($R_0 = 14$) nevertheless points to the great complexity and potentially significant heterogeneity of measles transmission and mixing contact patterns within the state. In addition, our modeling approach allowed us to estimate a measles CFR between 0.40% and 1.80% in India, which offers a defensible range of CFRs compared with those previously reported in the literature [7]. It also enabled us to quantify the burden of measles mortality in India, for which we derived estimates in the range of other studies [24, 25]. Lastly, our analysis confirmed the likely high impact [26–28] of measles SIAs in India which were rolled out for about 110 million Indian children aged 9 months to 10 years [20] and could have potentially averted 66,000 deaths [19]. This is also consistent with the significant child survival impact of SIA recently estimated for 25 sub-Saharan African countries [29].

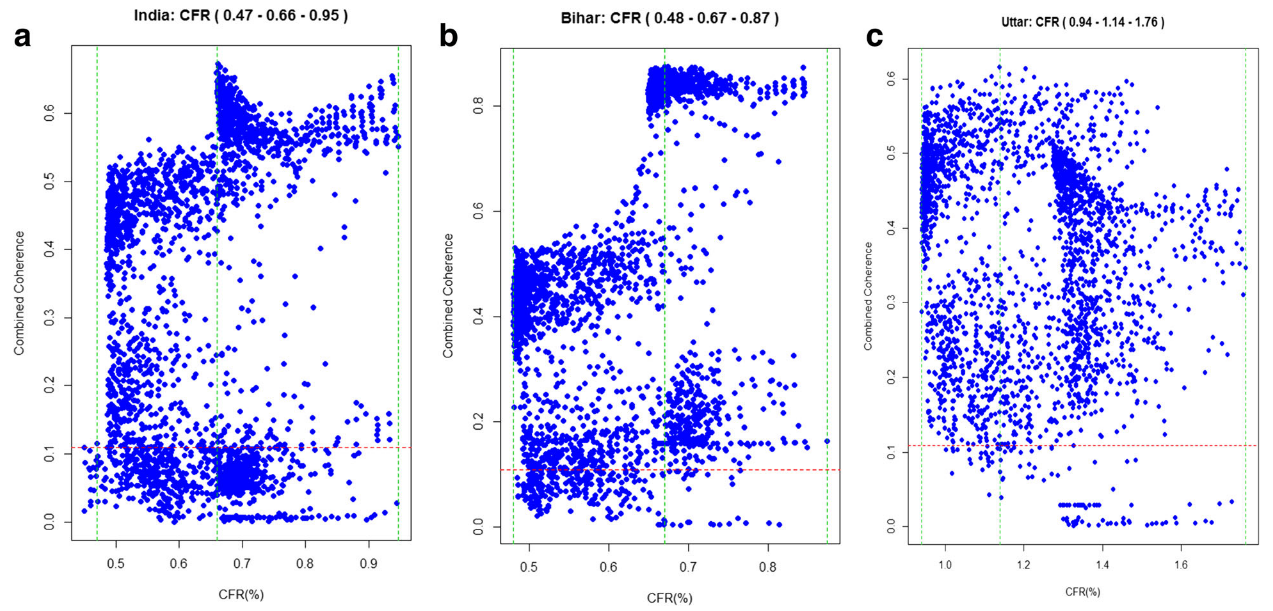

Fig. 3 Pooled coherence across five periods (0.5, 0.6, 0.75, 1.0, and 1.5 years) plotted against estimated case fatality risk (*CFR*) calculated from corresponding R_0 and a_0 for India (**a**), Bihar (**b**), and Uttar Pradesh (**c**). The *left* and *right green lines* delimit the range of the CFR where coherence is above the 95% significance level. The *middle green line* indicates the CFR for which the highest pooled coherence was estimated. The *bottom red line* indicates the upper 95% significance limit for the coherence function

Our modeling approach gives policymakers tools to estimate the impact of measles routine immunization and SIAs in India, Bihar, and UP. India is highly diverse in terms of geography, health systems, and local epidemiology. Due to a lack of robust mortality data at state and district levels, this analysis modeled an increase in overall vaccine coverage across India to the average level seen in the recent SIA, rather than the details of the SIA itself. As additional data become available, our model will be able to capture finer scale disease dynamics and more accurately assess intervention impact.

Our study presents a number of strengths and limitations. Our model is one of the few dynamic models [6, 30] of measles transmission calibrated to measles data from low- and middle-income countries; it is to the best of our knowledge the only such model calibrated to measles mortality data taking advantage of the spectral features (periodicity) of measles infection [31]. As such, it enables us to directly estimate the measles CFR for India, setting itself free from the vast uncertainty in measles CFRs pooled from previous studies [7]. In general, most attempts at estimating measles mortality and vaccine impact in low- and middle-income countries, and especially in India, have focused on assuming a measles CFR taken from the literature [5, 6, 27, 28, 32] and on good reporting of measles case notifications during outbreaks, which is unlikely. Hence, our paper proposes a new approach that could strengthen measles mortality estimates and also be applied to other settings where historical time series of measles cases or deaths are available. It also builds on a rich mortality dataset [8] rather than on under-reported case notifications [33]. Yet, it has a number of limitations. First, the time series used was short, with only 3 years of measles deaths data, which increased the uncertainty of the spectral estimation. Hence, the longest cycles in the spectrum estimated (with period 1.5 years) may actually be even longer (e.g., 2 years). This is supported by the fact that most measles epidemics observed in the real world have integer-valued periods. Other datasets may present a longer time period; however, they often have a much coarser nature (e.g., WHO measles case notification data [33]), such as annual data rather than weekly data as with the MDS. This can thus be equally restrictive in the periods calculable with Fourier analysis (Additional file 1: Web appendix III, section 4). Second, it is possible that some measles deaths were not recognized as such in the MDS study, and as a result our CFR estimation would correspond to a lower bound because of such under-ascertainment. However, the MDS dataset has been well validated and scrutinized to reduce biases and misclassifications in the assessment of under-five deaths [9, 10], so we would anticipate this under-ascertainment to be minimal. Third, small number issues prevented us from examining additional age groups (above age 5) and additional states besides the highly populated states of Bihar and UP. Fourth, our mortality data dated back from 2000 through 2003, which suggests that our extrapolation into the future should be interpreted with caution. For example, the CFR could well decrease over time as under-five mortality decreases and health services improve. Fifth, our modeling assumption of equilibrium behavior is a simplification, because when vaccine coverage and birth rates are changing, transitions in measles epidemic cycles can also occur [31, 34]. However, this assumption may not be highly inaccurate, because measles coverage in India has been relatively stable in 2000–2003, and the crude birth rate has been only gradually decreasing from 1990 to 2005 [35, 36].

Conclusions

~~India, one of the 47 priority countries where the measles burden is highest, represents a key country for measles elimination objectives. To accelerate its measles control efforts, between 2010 and 2013 India delivered a second opportunity for measles-containing vaccine through mass vaccination campaigns targeting states where access to health services is often weak.~~ Our modeling approach enables us to project measles incidence and mortality into the future, allowing us to assess whether India could reach measles elimination goals through both the scale-up of routine immunization and the implementation of mass campaigns. A similar approach (e.g., Fourier analysis) could be applied in any setting with a time series of measles deaths or notifications indicating the periodicity of outbreaks, regardless of under-reporting in the data. Hence, one could examine the progress of other measles priority countries toward achievement of measles control and elimination.

Additional file

Additional file 1: Web appendix I: Million Death Study research ethics. Web appendix II: Model structure. Web appendix III: Parameter estimation. ~~(DOC [illegible] kb)~~

Abbreviations

CFR: Case fatality risk; DynaMICE: Dynamic Measles Immunization Calculation Engine; MDS: Million Death Study; SIA: Supplemental immunization activity; UP: Uttar Pradesh; WHO: World Health Organization

Acknowledgements

This work was funded by the WHO's Initiative for Vaccine Research and Gavi, the Vaccine Alliance. The views expressed are those of the authors and do not necessarily represent the views of these institutions. This study could not have been completed without the support of Raymond Hutubessy. We thank Arindam Nandi and Ashvin Ashok for sourcing relevant data. We received valuable comments from Matthew Ferrari and from participants of a workshop on the broader economic benefits of vaccination and of the fourth meeting of WHO's SAGE working group in measles and rubella, particularly John Edmunds and Peter Strebel. Finally, we thank two reviewers for very helpful and constructive comments on our manuscript.

Availability of data and materials

Part of the data utilized in this study are secondary data extracted from a dataset property of the Registrar General of India and the overall mortality results have been published [10, 37]~~: application for dataset access can be made to the Office of the Registrar General of India.~~ The rest of the data utilized is available as referenced in the article.

Authors' contributions

SV and MJit initiated and conceptualized the study. WS and PJ provided the data. EJ, MJit, and SV coded the model and conducted the coherence analyses. SV coordinated the research and did the spectral analysis with EJ and MJit. SV wrote the first draft of the manuscript. MJit, EJ, MJohri, SKM, CLG, WS, and PJ reviewed the report and provided advice and suggestions. All authors read and approved the final manuscript.

Ethics approval and consent to participate

Anonymous secondary data were used (Additional file 1: Web appendix I provides MDS research ethics details).

Competing interests

The authors declare that they have no competing interests.

Publisher's Note

Springer Nature remains neutral with regard to jurisdictional claims in published maps and institutional affiliations.

Author details

[1]Department of Global Health and Population, Harvard T.H. Chan School of Public Health, 665 Huntington Avenue, Boston, MA, USA. [2]Department of Infectious Disease Epidemiology, London School of Hygiene and Tropical Medicine, London, UK. [3]University of Montreal Hospital Research Centre (CRCHUM), Montréal, Québec, Canada. [4]Department of Health Management, Evaluation and Policy, School of Public Health, University of Montreal, Montréal, Québec, Canada. [5]Division of Infectious Diseases, Hospital for Sick Children, Department of Pediatrics, University of Toronto, Toronto, Ontario, Canada. [6]Center for Global Health Research, Saint Michael's Hospital and University of Toronto, Toronto, Ontario, Canada. [7]Canadian Partnership Against Cancer, Toronto, Ontario, Canada. [8]Modelling and Economics Unit, Public Health England, London, UK.

Received: 16 December 2016 Accepted: 3 July 2017

References

1. World Health Organization. Global Immunization Vision and Strategy, 2006–2015. Geneva: World Health Organization; 2005. http://apps.who.int/iris/bitstream/10665/69146/1/WHO_IVB_05.05.pdf.
2. World Health Organization. Global Measles and Rubella Strategic Plan 2012–2020. Geneva: World Health Organization; 2012. http://www.measlesrubellainitiative.org/wp-content/uploads/2013/06/Measles-Rubella-Strategic-Plan.pdf.
3. Orenstein WA, Hinman AR, Strebel PJ. Measles: the need for 2 opportunities for prevention. Clin Infect Dis. 2006;42:320–1.
4. World Health Organization. Measles vaccines: WHO position paper. Wkly Epidemiol Rec. 2009;84:349–60.
5. Wolfson LJ, Strebel PM, Gacic-Dobo M, et al. Has the 2005 measles mortality reduction goal been achieved? A natural history modelling study. Lancet. 2007;369:191–200.
6. Simons E, Ferrari M, Fricks J, Wannemuehler K, Anand A, et al. Assessment of the 2010 global measles mortality reduction goal: results from a model of surveillance data. Lancet. 2012;379(9832):2173–8.
7. Wolfson LJ, Grais RF, Luquero FJ, Birmingham ME, Strebel PM. Estimates of measles case fatality ratios: comprehensive review of community-based studies. Int J Epidemiol. 2009;38:192–205.
8. Centre for Global Health Research. Million Death Study (MDS). http://www.cghr.org/index.php/projects/million-death-study-project. Accessed 29 Dec 2015.
9. Bassani DG, Kumar R, Awasthi S, Morris SK, Paul VK, et al. Causes of neonatal and child mortality in India: a nationally representative mortality survey. Lancet. 2010;376(9755):1853–60.
10. Morris SK, Awasthi S, Kumar R, Shet A, Khera A, et al. Measles mortality in high and low burden districts of India: estimates from a nationally representative study of over 12,000 deaths. Vaccine. 2013;31(41):4655–61.
11. Bracewell R. The Fourier transform and its applications. New York: McGraw-Hill; 1965.
12. Ram U, Jha P, Ram F, Kumar K, Awasthi S, et al. Neonatal, 1-59 month, and under-5 mortality in 597 Indian districts, 2001 to 2012: estimates from national demographic and mortality surveys. Lancet Global Health. 2013;1(4):e219–26.
13. Government of India Ministry of Health and Family Welfare. 2009 coverage evaluation survey. New Delhi: UNICEF India Country Office; 2010.
14. Verguet S, Johri M, Morris SK, Gauvreau CL, Jha P, Jit M. Controlling measles using supplemental immunization activities: a mathematical model to inform optimal policy. Vaccine. 2015;33:1291–6.
15. Mossong J, Hens N, Jit M, Beutels P, Auranen K, et al. Social contacts and mixing patterns relevant to the spread of infectious diseases. PLoS Med. 2008;5(3):e74.
16. Sudfeld CR, Navar AM, Halsey NA. Effectiveness of measles vaccination and vitamin A treatment. Int J Epidemiol. 2010;39 Suppl 1:48–55.
17. Office of the Registrar General & Census Commissioner, India. Census of India 2001. Population projections for India and states 2001–2026 (revised December 2006).
18. Anderson RM, May RM. Infectious diseases of humans: dynamics and control. Oxford: Oxford University Press; 1991.
19. Johri M, Verguet S, Morris SK, et al. Adding interventions to mass measles vaccinations in India. Bull World Health Organ. 2016;94:718-27.
20. Ministry of Health and Family Welfare India. Measles SIA Coverage India 2010–2013. New Delhi: Ministry of Health and Family Welfare India; 2014.
21. Baxter M, King RG. Measuring business cycles: approximate band-pass filters for economic time series. Rev Econ Stat. 1999;81(4):575–93.
22. Brillinger DR. Time series: data analysis and theory. 2nd ed. San Francisco: Holden Day; 1981.
23. Amjad AM, Halliday DM, Rosenberg JR, Conway BA. An extended difference of coherence test for comparing and combining several independent coherence estimates: theory and application to the study of motor units and physiological tremor. J Neurosci Methods. 1997;73(1):69–79.
24. Institute for Health Metrics and Evaluation. Global Burden of Disease Study 2013. http://vizhub.healthdata.org/gbd-compare/. Accessed 30 Dec 2015.
25. Liu L, Oza S, Hogan D, Perrin J, Rudan I, et al. Global, regional, and national causes of child mortality in 2000–2013, with projections to inform post-2015 priorities: an updated systematic analysis. Lancet. 2015;385:430–40.
26. Dabral M. Cost-effectiveness of supplementary immunization for measles in India. Indian Pediatr. 2009;46:957–62.
27. Bishai D, Johns B, Lefevre A, Nair D. Cost-effectiveness of measles eradication. Final report. http://www.who.int/immunization/sage/1_Bishai_Economic_analysis.pdf. Accessed 29 Dec 2015.
28. Levin A, Burgess C, Garrison L, Bauch C, Babigumira J. Economic evaluation of measles eradication study: results for six countries and by income groups. http://www.who.int/immunization/sage/2_Levin_Economic_Evaluation_of_Measles_Eradication_final.pdf. Accessed 17 Aug 2010.
29. Ben Yishai A, Kranker K. All-cause mortality reductions from measles catchup campaigns in Africa. J Hum Resour. 2015;50(2):516–47.
30. Ferrari MJ, Grais RF, Bharti N, Conlan AJK, Bjornstadt O, et al. The dynamics of measles in sub-Saharan Africa. Nature. 2008;451:679–84.
31. Earn DJ, Rohani P, Bolker B, Grenfell BT. A simple model for complex dynamical transitions in epidemics. Science. 2000;287:667–70.
32. Ozawa S, Clark S, Portnoy A, Grewal S, Brenzel L, Walker DG. Return on investment from childhood immunization in low- and middle-income countries, 2011–20. Health Affairs. 2016;35(2):199–207.
33. World Health Organization. Measles surveillance data. http://www.who.int/immunization/monitoring_surveillance/burden/vpd/surveillance_type/active/measles_monthlydata/en/. Accessed 29 Dec 2015.
34. Hempel K, Earn DJD. A century of transitions in New York City's measles dynamics. J R Soc Interface. 2015;12:20150024.
35. World Health Organization. WHO-UNICEF estimates of MCV1 coverage. http://apps.who.int/immunization_monitoring/globalsummary/timeseries/tswucoveragemcv1.html. Accessed 19 Mar 2017.
36. World Bank. World Development Indicators. http://data.worldbank.org/data-catalog/world-development-indicators. Accessed 19 Mar 2017.
37. Registrar General of India and Center for Global Health Research. Causes of death in India, 2001-2003: sample registration system. Government of India; 2009.
